# In Situ-Targeting of Dendritic Cells with Donor-Derived Apoptotic Cells Restrains Indirect Allorecognition and Ameliorates Allograft Vasculopathy

**DOI:** 10.1371/journal.pone.0004940

**Published:** 2009-03-31

**Authors:** Zhiliang Wang, William J. Shufesky, Angela Montecalvo, Sherrie J. Divito, Adriana T. Larregina, Adrian E. Morelli

**Affiliations:** 1 Thomas E. Starzl Transplantation Institute, University of Pittsburgh Medical Center, Pittsburgh, Pennsylvania, United States of America; 2 Department of Surgery, University of Pittsburgh Medical Center, Pittsburgh, Pennsylvania, United States of America; 3 Department of Dermatology, University of Pittsburgh Medical Center, Pittsburgh, Pennsylvania, United States of America; 4 Department of Immunology, University of Pittsburgh Medical Center, Pittsburgh, Pennsylvania, United States of America; New York University School of Medicine, United States of America

## Abstract

Chronic allograft vasculopathy (CAV) is an atheromatous-like lesion that affects vessels of transplanted organs. It is a component of chronic rejection that conventional immuno-suppression fails to prevent, and is a major cause of graft loss. Indirect allo-recognition through T cells and allo-Abs are critical during CAV pathogenesis. We tested whether the indirect allo-response and its impact on CAV is down-regulated by in situ-delivery of donor Ags to recipient's dendritic cells (DCs) in lymphoid organs in a pro-tolerogenic fashion, through administration of donor splenocytes undergoing early apoptosis. Following systemic injection, donor apoptotic cells were internalized by splenic CD11c^hi^ CD8α^+^ and CD8^−^ DCs, but not by CD11c^int^ plasmacytoid DCs. Those DCs that phagocytosed apoptotic cells in vivo remained quiescent, resisted ex vivo-maturation, and presented allo-Ag for up to 3 days. Administration of donor apoptotic splenocytes, unlike cells alive, (i) promoted deletion, FoxP3 expression and IL-10 secretion, and decreased IFN-γ-release in indirect pathway CD4 T cells; and (ii) reduced cross-priming of anti-donor CD8 T cells in vivo. Targeting recipient's DCs with donor apoptotic cells reduced significantly CAV in a fully-mismatched aortic allograft model. The effect was donor specific, dependent on the physical characteristics of the apoptotic cells, and was associated to down-regulation of the indirect type-1 T cell allo-response and secretion of allo-Abs, when compared to recipients treated with donor cells alive or necrotic. Down-regulation of indirect allo-recognition through in situ-delivery of donor-Ag to recipient's quiescent DCs constitutes a promising strategy to prevent/ameliorate indirect allorecognition and CAV.

## Introduction

Chronic rejection (CR) is a major problem in transplantation that immuno-suppressive reagents fail to prevent and that limits long-term allograft survival [1]. One of the key features of CR is the development of chronic allograft vasculopathy (CAV), a condition manifested by endothelialitis, intimal thickening, elastic fiber disruption, adventitial fibrosis and leukocyte infiltration of arteries of the graft [2]. As a consequence, the vessels undergo progressive reduction of their lumen and become susceptible to thrombosis [2]. CAV results form non-immune and immune mechanisms, and lesions similar to CAV have been reproduced in experimental models by adoptive transfer of allo-Abs or donor-reactive PBMCs [3]–[5]. More recently, it has been shown that cells of the innate immune system, including NK cells [6], [7] and macrophages [8] participate in the pathogenesis of CAV.

T cell recognize donor allo-peptides presented by recipient's APCs through the indirect pathway of allorecognition [9], a mechanism that generates indirect pathway CD4 T cells and allo-Abs, both critical in the pathogenesis of CAV [10]–[12] and CR [13], [14]. By using mice deficient in dendritic cells (DCs), we have previously shown that recipient's DCs are instrumental for development of CAV [15]. However, besides their role in T cell immunity, DCs resident in secondary lymphoid organs are critical for induction of peripheral T cell tolerance in the steady-state [16], a property that could be exploited therapeutically to restrain the indirect alloresponse that leads to CAV [17].

The classic tolerogenic DC-based methods to down-regulate the anti-donor response and prolong allograft survival are based on administration of donor- or recipient-derived tolerogenic DCs generated in vitro [reviewed in 18]. This first generation of DC-therapies for transplantation has the following limitations for its clinical application: (i) the time required for in vitro-generation of DCs results a complication when the DCs have to be prepared from deceased donors; (ii) the maturation of the injected DCs may sensitize the recipient; (iii) injected DCs are re-processed by recipient's APCs; (iv) donor-derived DCs are targets of recipient's NK cells [19]; and (v) only a small percentage of the injected DCs home in secondary lymphoid organs.

An alternative DC-based method to control the indirect alloresponse that leads to CAV consists in “in situ-delivery” of donor allo-Ags to recipient's DCs resident in secondary lymphoid organs [17], [18], without perturbing the quiescent state of the APCs [20]. We and others [21]–[23] have shown that systemic administration of leukocytes undergoing apoptosis constitutes and efficient method to deliver foreign Ags to DCs in vivo, without inducing DC activation. This is because cells in early apoptosis (i) are processed efficiently by DCs and (ii) exert regulatory effects on APCs [21]–[31]. Indeed, the apoptotic cell clearance resulting from the physiological cell turnover in peripheral tissues by DCs plays a key role in peripheral T cell tolerance [32]. These observations have led to the recent development of apoptotic cell-based strategies to promote immunologic tolerance for therapy of transplantation and autoimmunity. However, the mechanisms of action of apoptotic cell-based therapies in vivo, their effects on different DC subsets in vivo, and their therapeutic potential on chronic rejection (currently one of the main problems in transplantation) have not been elucidated.

In this study, we tested whether is situ delivery in a pro-tolerogenic fashion of donor allo-Ags to recipient's DCs by therapy with apoptotic cells, down-regulates the indirect anti-donor response and its effects on CAV. We characterized the DC subsets targeted in vivo by this methodology and its impact on the DCs and on allo-Ag presentation to indirect pathway T cells. Our findings demonstrate that the features of CAV in a mouse model of fully-mismatched aortic allografts (an established model of CAV), are clearly ameliorated by in situ-targeting of DCs with donor allo-Ags, and correlate with the ability of apoptotic cells, unlike alive or necrotic cells, to restrain efficiently the anti-donor indirect T and B cell responses in allograft recipients.

## Materials and Methods

### Mice and reagents

Ten-12-wk-old C57BL/6 (B6, H2^b^), BALB/c (H2^d^), C3H (H2^k^), B6×BALB/c (F1), β_2 m_^−/−^ B6, and B6.FVB-Tg (Itagx-DTR/eGFP)_57_Lan/J (CD11c-DTR-eGFP, H2^b^) mice were from The Jackson Laboratory. 1H3.1 and 2C*Rag1^−/−^* TCRtg mice were provided by Dr. C. Viret (Yale University) and Dr. G. Chalasani (Univ. of Pittsburgh). Studies were approved by the IACUC. FGK4.5 mAb was purchased from BIOExpress. PKH67, histodenz™, LPS, and diphtheria toxin (DT) were from Sigma. The apoptosis detection kit was from BD Pharmingen. Mouse rGM-CSF was from Peprotech. The IEα_52–68_ (ASFEAQGALANIAVDKA) and SYGL (SIYRYYGL) peptides were synthesized, HPLC-purified and confirmed by mass spectroscopy.

### Generation of apoptotic and necrotic cells

Apoptotic splenocytes were generated by 3 min UV-B irradiation with an UVB lamp (ULTRA-LUM, Claremont, CA, model UVB16) [23]. The percentage of cells in early apoptosis (annexin V^+^, propidium iodide^−^) reached 90–95% 3 h after irradiation, with less than 5–10% of cells in late apoptosis. Necrotic splenocytes were generated by 1 freeze-thaw cycle. BALB/c splenocytes were stained with PKH67 before UV-irradiation or freezing-thawing.

### Purification and labeling of DCs

Spleens and thymi from B6 mice, untreated or injected with PKH67^+^ apoptotic or necrotic BALB/c splenocytes, were flushed with 100 U/ml collagenase and digested with 400 U/ml collagenase (30 min, 37°C). Cells were resuspended in cold Ca-free 0.01 M EDTA/HBSS and centrifuged over 16% histodenz gradient. Splenic DC-enriched suspensions (20–30% DCs) were blocked with 10% normal goat serum and incubated with the following mAbs (BD Pharmingen, eBioscience): i) CyChrome (CyC)-CD11c; ii) PE-H2K^b^, -IA^b^, -CD40, -CD45RA, -CD80, -CD86, -ICOSL, -CD178 (FasL), -PD-L1 or -PD-L2; and iii) APC-CD8α. Species- and isotype-matched Igs were used as controls. Cells were fixed in 2% paraformaldehyde and analyzed with a BD FACSCalibur™ flow cytometer (BD Biosciences). Liver nonparenchymal cells were purified as described [33].

### Adoptive transference of TCRtg T cells

1H3.1 CD4 T cells (Thy1.1 congenic) were purified from spleens and lymph nodes by depleting CD8, B220, IA^b^, F4/80 and NK1.1-expressing cells (Dynabeads®). 1H3.1 cells were labeled or not with 5 µM CFSE (Molecular Probes) and 3×10^6^ cells were administered i.v. to WT or CD11c-DTR-eGFP B6 mice. In some experimental groups, mice were injected with DT (4 ng/g body weight, i.p.). One day later, animals received i.v. 10^7^ BALB/c splenocytes alive or apoptotic, the latter alone or with agonistic CD40 mAb (FGK4.5; 150 µg; i.p.; on days 1–3). As control, B6 mice were injected with 10^7^ B6 apoptotic splenocytes alone. Three and 14 days later, cells from spleens and lymph nodes (inguinal, axilar, cervical, mesenteric) were stained with the following mAbs: i) CyC-CD4; ii) PE-CD62L, -CD69 or -FoxP3; and iii) APC-Thy1.1. Species- and isotype-matched Igs were used as controls. Cells were fixed in 2% paraformaldehyde and FACS-analyzed.

### In vivo killing assay

B6 (H2K^b^) mice, reconstituted with 2C CD8 T cells (2×10^6^ cells/ i.v.), were treated (i.v.) the following day with apoptotic or alive β_2 m_^−/−^ B6 splenocytes pre-loaded (or not, control) in vitro with the SYGL peptide (1 µg/ml, 3 h, 37°C). Six days later, mice were challenged (or not, control) with 25 µg of SYGL peptide in incomplete Freund adjuvant (IFA, 200 µl, i.p.). Three days later, mice received a 1∶1 mixture of target B6×BALB/c splenocytes labeled with 5 µM CFSE (CFSE^hi^), and control B6 splenocytes labeled with 0.5 µM CFSE (CFSE^lo^) (2×10^7^ total cells in 300 µl PBS / mouse). Five h later, the percentages of splenic CFSE^hi^ and CFSE^lo^ cells was assessed by FACS. Percent specific lysis was calculated by the formula: {1−[(ratio of CFSE^lo^/CFSE^hi^ of naive mouse) / (ratio of CFSE^lo^/ CFSE^hi^ of challenged mouse)]}×100.

### ELISPOT and allo-Ag proliferation assays

For ELISPOT analysis of 1H3.1 T cells, splenocytes of host B6 mice [reconstituted with 1H3.1 CD4 T cells and then treated with BALB/c (or B6, control) apoptotic, necrotic or living splenocytes] were incubated with (or without, control) 1 µg/ml IEα_52–68_, in 96-well ELISPOT plates (BD Biosciences) coated with IFN-γ, IL-4, IL-10, IL-17 or TGF-β1 mAbs (2.5×10^5^ cells/ well). ELISPOT plates were cultured for 36 h followed by incubation with biotin-IFN-γ, -IL-4, -IL-10, -IL-17 and -TGF-β1 mAbs, streptavidin-peroxidase and 3-amino-9-ethylcarbazole (AEC). The spots were counted with an ImmunoSpot™ counter (Cellular Technology Ltd., Cleveland, OH). For allo-Ag proliferation assays of 1H3.1 T cells, splenocytes were incubated with (or without, control) 1 µg/ml IEα_52–68_ in round-bottom 96-well plates (2.5×10^5^ cells /well). Cell proliferation was evaluated 72 h later by assessment of [^3^H]thymidine incorporation.

For ELISPOT analysis of the indirect response in graft recipients, purified splenic T cells (enrichment columns, R&D Systems) from B6 mice transplanted with syngeneic or BALB/c aortas were incubated with CD3-depleted, γ-irradiated, splenic B6 APCs (3×10^5^ T cells and 2.5×10^6^ APCs / well) and 20 µl of sonicates prepared from BALB/c, B6 or C3H splenocytes, in 96-well ELISPOT plates coated with IFN-γ mAb [34]. For ELISPOT analysis of the direct response, purified splenic T cells (enrichment columns, R&D Systems) from B6 mice transplanted with syngeneic or BALB/c aortas were incubated with CD3-depleted, γ-irradiated, splenic BALB/c, B6 or C3H APCs (5×10^4^ T cells and 2.5×10^5^ APCs / well) in 96-well ELISPOT plates coated with IFN-γ mAb.

### Aortic transplantation

Intra-abdominal aortic transplantation was performed as described [35]. A 6–10 mm segment of the donor's descending thoracic aorta was anastomosed (end to side) to the recipient's abdominal aorta. The native abdominal aorta was then ligated and severed, converting the end-to-side anastomosis to a quasiend-to-end anastomosis.

### Analysis of aortic grafts

Aortic grafts were harvested 1 mm beyond the sutures and divided into two equal segments. One fragment was fixed in 4% paraformaldehyde, processed and cross-sectioned for staining with H&E, Verhoeff-van Gieson's (elastic fibers), Masson's trichrome (collagen) and immunoperoxidase for α-smooth muscle (α-sm) actin^+^ cells. Sections were blocked with 10% normal goat serum and incubated with α-sm actin mAb (Dako-1A4), biotin- secondary Ab and the final product visualized with the ABC immunoperoxidase system with the substrate AEC (Vector). The other half of the graft was embedded in Tissue-Tek OCT (Miles Laboratories), snap-frozen, and stored at −80°C. Cryostat sections (6 µm) were air-dried, fixed in 96% ethanol, and blocked with 10% normal goat serum and the avidin/biotin blocking kit (Vector). For detection of CD4 together with CD8 or FoxP3, sections were incubated with alexa 488-CD4 and biotin-CD8 or biotin-FoxP3 mAbs followed by Cy3-streptavidin. For co-detection of IgG and C3d, sections were incubated with 2-fold dilutions (1∶25–1∶400) of alexa 488 anti-mouse IgG (Molecular Probes) and biotin C3d (1∶100) mAbs (R&D), followed by streptavidin-Cy3. Nuclei were stained with DAPI (Molecular Probes). IgG deposition was assessed semi-quantitatively by determination of the end-titer dilution of the anti-mouse IgG mAb. Sections were analyzed with a fluorescence microscope (Zeiss) equipped with an imaging system and the image analyzing software Axio Vision.

### Detection of alloantibodies

Sera allo-Abs were FACS-analyzed in naïve B6 and in B6 mice transplanted with syngeneic or BALB/c aortas. BALB/c splenocytes were incubated with a 1∶10 dilution of de-complemented sera. Cells were then incubated with PE-CD19 mAb and one of the following FITC-mAbs: i) F(ab′)_2_ anti-mouse Fcγ (Jackson ImmunoResearch); ii) anti-mouse IgG_1_; or iii) anti-mouse IgG2_a_ (BD Pharmingen).

### Statistical analyses

Results are expressed as means±SD. Comparisons between more than 2 means were performed by ANOVA, followed by the Student Newman Keuls test. Comparison between two means was performed by Student's “t” test. A “p” value<0.05 was considered significant.

## Results

### Donor apoptotic cells administered systemically are presented by CD11c^hi^ DCs

The following subsets of DCs coexist in mouse secondary lymphoid organs: i) CD11c^hi^CD8^−^ DCs (myeloid); ii) CD11c^hi^CD8α^+^ DCs (lymphoid-related) and iii) CD11c^int^CD45RA^+^ B220^+^Gr1^+^ plasmacytoid DCs (pDCs) [36]. We and others have shown in mice that (i) blood-borne leukocytes undergoing apoptosis are captured by splenic CD11c^hi^ DCs [21]–[23], and (ii) in situ-delivery of allo-Ags to splenic DCs by i.v. injection of donor apoptotic cells down-regulates anti-donor T cell immunity, prolongs cardiac allograft survival [37], [38] and facilitates BM engraftment [39]–[41]. Although CD11c^int^ pDCs also play an important role in down-regulating the T cell response [42]–[44] and likely in transplantation tolerance [45]–[47], their ability to interact with apoptotic cells is still controversial [48]–[51]. Thus, we assessed in vivo the ability of recipient's pDCs to capture donor apoptotic cells administered systemically, and the role of pDCs in the beneficial effect of apoptotic cell therapy in transplantation.

**Figure 1:**
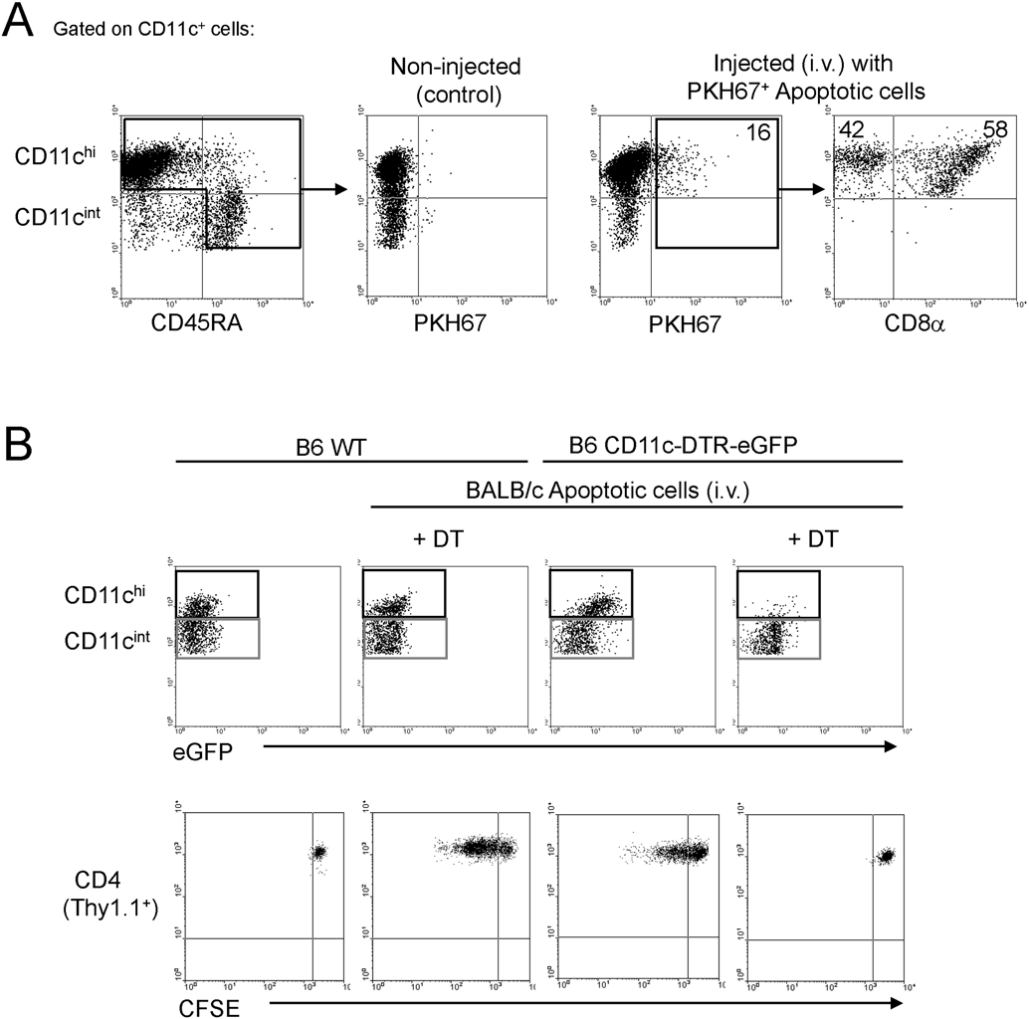
Blood-borne donor apoptotic cells are captured and presented to T cells by splenic CD11c^hi^ DCs. A) PKH67-labeled BALB/c apoptotic cells were injected i.v. in B6 mice and the entrapment of blood-borne PKH-67^+^ (green) apoptotic cell fragments by splenic CD11c^hi^ CD45RA^−^ DCs (CD8α^+^ or CD8α^−^) and CD11c^int^ CD45RA^+^ pDCs was analyzed by FACS, 18 h later. B) WT and CD11c-DTR-eGFP B6 mice (both Thy1.2^+^) were reconstituted with CFSE-labeled 1H3.1 CD4 T cells (Thy1.1^+^), then treated (or not, control) with DT and injected i.v. with BALB/c apoptotic cells. Injection of DT in CD11c-DTR-eGFP mice deleted selectively CD11c^hi^ DCs and spared CD11c^int^ pDCs in the spleen (upper dot plots). In the absence of splenic pDCs, CD11c-DTR-eGFP mice were unable to present BALB/c apoptotic cell-derived allopeptides and induce proliferation of the adoptively transferred 1H3.1 CD4 T cells, evaluated 48 h later by CFSE-dilution by FACS-analysis (bottom dot plots). One representative out of 5 (in A) and 3 (in B) individual experiments is shown.

To address this question, PKH67-labeled (green) UVB-induced early apoptotic BALB/c splenocytes were injected i.v. in B6 mice (10^7^cells / mouse). Since the highest percentages of splenic DCs with internalized allogeneic apoptotic cells are detected 18–24 h after i.v. injection [23], we analyzed by FACS the percentages of splenic DC subsets containing PKH67^+^ apoptotic cell fragments, 18 h after apoptotic cell administration. As expected [23], 18±6% of CD11c^hi^ DCs (60±4% CD8α^+^ and 39±6% CD8α^−^ DCs) contained PKH67^+^ fragments (Fig. 1A). By contrast, we did not detect PKH67^+^ content within CD11c^int^ CD45RA^+^ pDCs (Fig. 1A). Less than 1% of CD11c^hi^ DCs and no pDCs from lymph nodes, thymus and liver internalized the injected apoptotic cells (not shown). Necrotic PKH67^+^ BALB/c splenocytes were very poorly or no internalized by spleen, thymus, lymph node or liver CD11c^hi^ DCs (≤2%) or CD11c^int^ pDCs (≤0.01%) (not shown).

To further confirm that pDCs were unable to process and present Ags derived from donor apoptotic cells, we tested if B6 splenic pDCs present the BALB/c apoptotic cell-derived allopeptide IEα_52–68_ to CFSE-labeled Thy1.1 congenic 1H3.1 TCRtg CD4 T cells [specific for IA^b^ (from B6)-IEα_52–68_, 52]. To assess the role of pDCs, we used as hosts B6 CD11c-DTR-eGFP mice (Thy1.2 congenic), a DT receptor (DTR)-based system where CD11c^hi^ DCs can be conditionally ablated for up to 2–3 days by administration of DT [53], whereas CD11c^int^ pDCs are spared from deletion [54]. Forty eight h after injection of BALB/c apoptotic cells into B6 CD11c-DTR-eGFP mice, we detected proliferation of splenic 1H3.1 cells, assessed by CFSE-dilution analyzed by FACS (Fig. 1B). B6 CD11c-DTR-eGFP mice treated with DT exhibited almost complete deletion of CD11c^hi^ DCs with preservation of CD11c^int^ pDCs and no proliferation of splenic 1H3.1 cells (Fig. 1B). The lack of division of 1H3.1 cells was not due to the DT, since 1H3.1 cells proliferated in WT B6 mice injected with BALB/c apoptotic cells and DT (Fig. 1B). These findings indicate that only splenic CD11c^hi^ DCs capture and process blood-borne donor apoptotic cells for presentation to indirect CD4 T cells.

### DCs that internalize apoptotic cells in vivo remain quiescent and resistant to maturation

**Figure 2:**
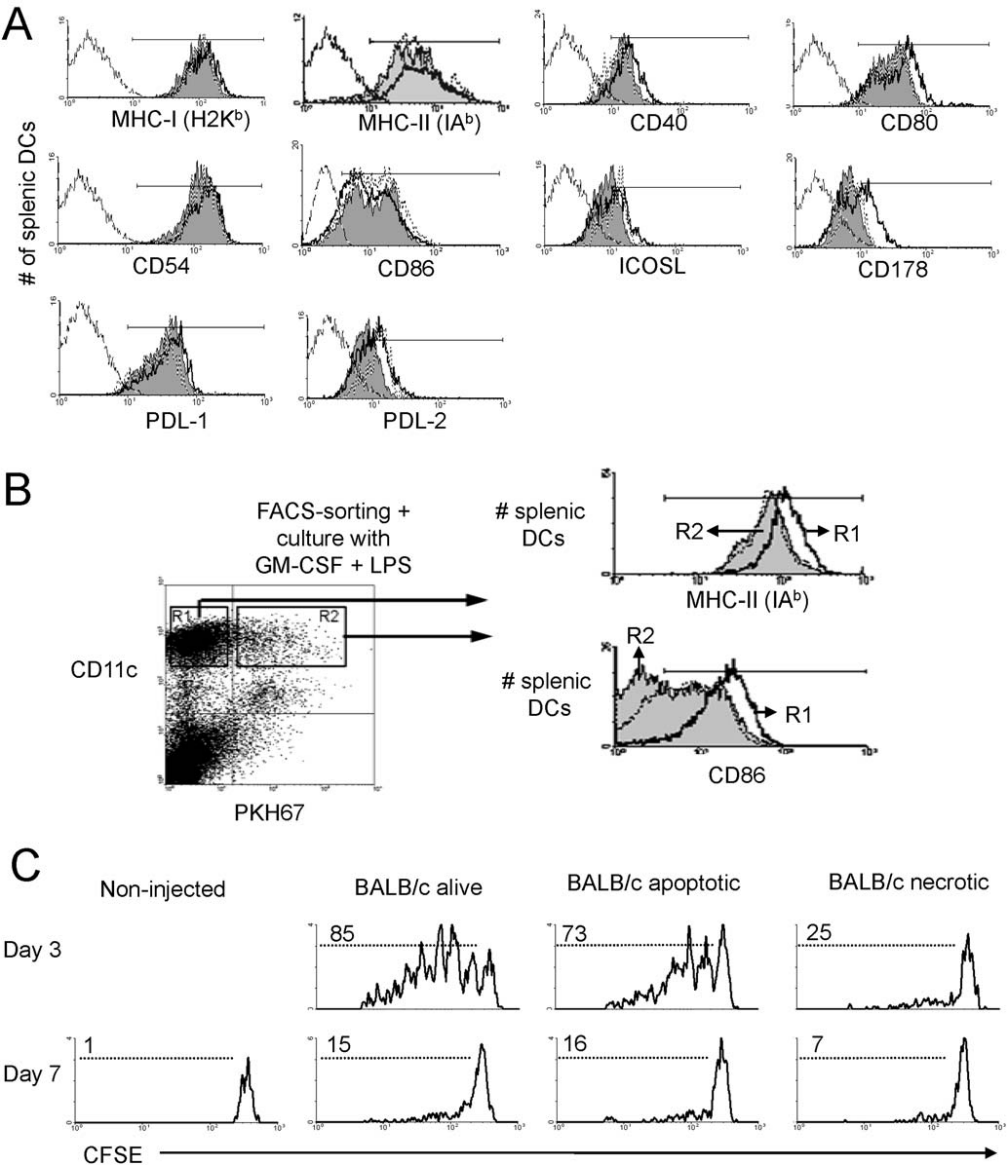
Splenic DCs remain quiescent following interaction with donor apoptotic cells in vivo. A) B6 mice were left untreated or injected i.v. with PKH67-labeled BALB/c apoptotic splenocytes. Histograms show the expression of surface markers by splenic CD11c^hi^ DCs (i) with internalized PKH-67^+^ apoptotic cell fragments (thick line), (ii) without PKH-67^+^ fragments from the same mice (gray), and (iii) from control non-injected mice (dotted line). One representative out of 3 individual experiments is illustrated. B) Eighteen h after i.v. administration of PKH-67-labeled BALB/c apoptotic splenocytes in B6 mice, splenic CD11c^hi^ DCs with internalized PKH-67^+^ fragments (R2) or not (R1) were FACS-sorted and cultured in medium with GM-CSF and LPS. After 24 h, surface expression of MHC class-II Ag and CD86 was analyzed by flow cytometry in the FACS-sorted splenic DCs that had internalized PKH-67^+^ fragments (gray histograms) or not (thick line histograms), and compared to the phenotype of freshly-isolated splenic CD11c^hi^ DCs (dotted line histogram). One representative out of 3 experiments is shown. C) CFSE-labeled 1H3.1 CD4 T cells were adoptively transferred (i.v.) into B6 mice at different times after administration of BALB/c splenocytes (alive, apoptotic or necrotic) and proliferation of 1H3.1 T cells was evaluated based on CFSE dilution assessed by FACS, 3 days after T cell transference. Presentation of BALB/c allopeptides to splenic 1H3.1 CD4 T cells (based on CFSE-dilution) decreased drastically by 7 days. Numbers represent percentages of dividing cells. One representative experiment with 3 mice per group and time point is shown.

There is evidence that DCs that internalize apoptotic cells fail to upregulate MHC Ag, CD40, CD80 and CD86 in vitro [23]–[30] and in vivo [37]. However, it is unknown whether this phenomenon also affects the expression of immune–regulatory molecules like PDL-1, PDL-2, ICOS-L and Fas-L. To address this, B6 mice were injected with PKH67^+^ BALB/c apoptotic cells and the phenotype of splenic CD11c^hi^ DCs was analyzed in DC-enriched suspensions by FACS, 6, 24 and 48 h later, time points when the DCs should be interacting with Ag-specific T cells. Internalization of PKH-67^+^ apoptotic cell fragments did not affect expression of MHC-I/II, CD40, CD54, CD86, PD-L1, PD-L2, and ICOS-L, and induced a modest up-regulation of CD80 and Fas-L (CD178) in splenic CD11c^hi^ DCs, compared with DCs without apoptotic cells from the same animal, or with DCs from non-injected controls (Fig. 2A, 24 h).

Next, we tested whether splenic CD11c^hi^ DCs that internalize donor apoptotic cells in vivo are resistant to maturation. Eighteen h after i.v. administration of PKH67^+^ BALB/c apoptotic cells in B6 mice, splenic CD11c^hi^ DCs that had internalized (PKH67^+^) or not (PKH67^−^) apoptotic cells were FACS-sorted and cultured for 24 h in medium with GM-CSF (1000 u/ml) and LPS (100 ng/ml) (Fig. 2B). Unlike control splenic DCs without apoptotic cells, DCs that had phagocytosed apoptotic cells in vivo (from the same mice) failed to up-regulate MHC-II Ag and CD86 ex vivo in response to overnight stimulation with LPS (or 5 µg/ml of agonistic CD40 mAb, not shown) (Fig. 2B). DCs with internalized apoptotic cells did not exert a bystander inhibition on the phenotype of those DCs that did not contain apoptotic cell fragments, since the latter DCs expressed levels of MHC-II Ag and CD86 comparable to those of splenic DCs freshly-isolated from control mice not treated with apoptotic cells (Fig. 2B). Thus, following internalization of apoptotic cells in vivo, splenic DCs remained quiescent, without altering their net balance of co-stimulatory/-regulatory molecules, failed to mature ex vivo, and did not exert bystander inhibitory effects on the phenotype of neighboring DCs without apoptotic cell fragments.

### Splenic DCs present apoptotic cell-derived allopeptides for a limited time-span

We assessed the duration of presentation of apoptotic cell-derived allopeptides by splenic DCs in B6 mice injected with BALB/c apoptotic cells, 28, 14, 7, 3 and 1 day prior to adoptive transference of CFSE-labeled 1H3.1 CD4 T cells (day 0). Three days after 1H3.1 cell transfer, proliferation of splenic 1H3.1 T cells was evaluated by FACS-analysis. As controls, B6 mice were injected i.v. with the same numbers of alive or necrotic BALB/c splenocytes. Presentation of the apoptotic cell-derived IEα_52–68_ allopeptide to 1H3.1 T cells reached a plateau 3 days after apoptotic cell administration, decreased by day 7 (Fig. 2C), and was undetectable thereafter. Although unlike apoptotic cells, necrotic cells promotes maturation of DCs [26], [27], we detected minimum proliferation of splenic 1H3.1 T cells in vivo following infusion of necrotic BALB/c splenocytes (Fig. 2C). This latter result can be explained by the fact that systemically administered necrotic cells were very poorly internalized by splenic DCs.

### Effect of in situ-targeting of splenic DCs on indirect pathway CD4 T cells

**Figure 3:**
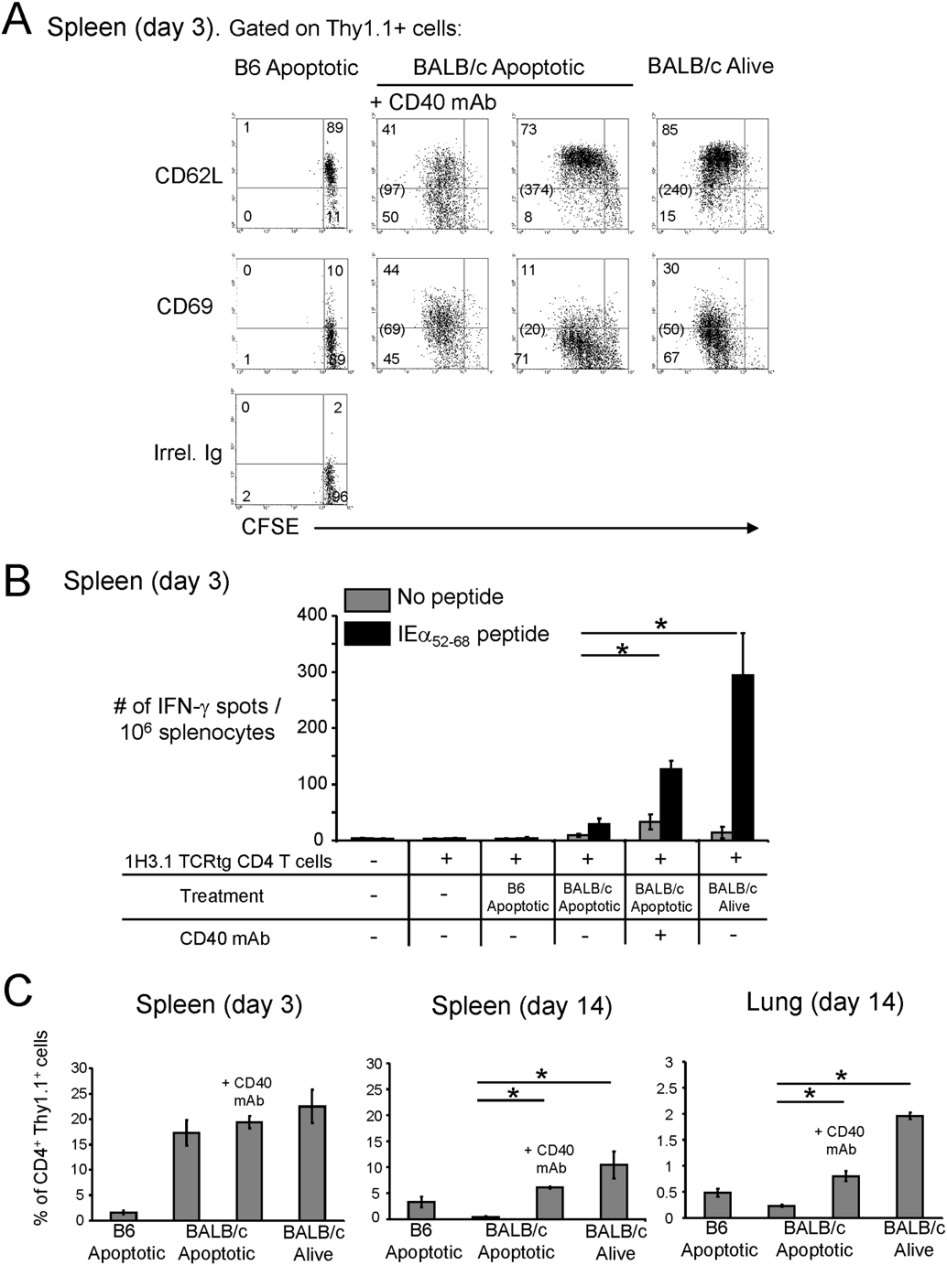
Donor apoptotic cells are recognized by indirect CD4 T cells. A) Analysis by FACS of proliferation and phenotype of CFSE-labeled 1H3.1 CD4 T cells transferred into B6 mice that were then treated (i.v.) with BALB/c splenocytes, alive or apoptotic. As control, to promote 1H3.1 T cell activation/proliferation, a group of B6 mice was treated with BALB/c apoptotic cells plus agonistic CD40 mAb. Numbers in dot plots represent percentages of cells. Numbers in parenthesis indicate the mean fluorescence intensity of the left quadrants combined. One representative experiment out of 6 is shown. B) ELISPOT analysis for IFN-γ of splenocytes from B6 mice reconstituted (or not, control) with 1H3.1 CD4 T cells and then injected i.v. with B6 (control) or BALB/c apoptotic splenocytes (alone or with agonistic CD40 mAb), or with BALB/c splenocytes alive. Three days later, splenocytes from the host B6 mice were cultured for 36 h in ELISPOT plates alone, or with the BALB/c peptide IEα_52–68_. Each group included 6 mice. C) Assessment by FACS of the percentages of CD4 1H3.1 T cells (Thy1.1^+^) in tissues of host B6 mice (Thy1.2^+^) previously reconstituted with 1H3.1 cells and then treated with B6 (control) or BALB/c apoptotic splenocytes (the latter alone or with agonistic CD40 mAb), or with BALB/c splenocytes alive. Results represented values pooled from 6 mice per group. * p<0.01.

We investigated whether in situ-targeting of DCs with donor apoptotic cells affects the anti-donor response elicited by indirect pathway CD4 T cells, and if the effect differs from that induced by donor splenocytes alive. B6 mice, reconstituted with CFSE-labeled naïve (CD62L^hi^ CD44^lo^) 1H3.1 CD4 T cells, were injected i.v. with BALB/c splenocytes alive or apoptotic the following day. As positive control of T cell activation, a group received BALB/c apoptotic cells plus agonistic CD40 mAb. Infusion of BABL/c splenocytes, alive or apoptotic, induced proliferation of splenic 1H3.1 cells assessed 3 days later by FACS-analysis (Fig. 3A). Unlike apoptotic cells, injection of cells alive was accompanied by division of 1H3.1 T cells in lymph nodes (not shown), a difference that can be explained because living cells migrate actively through all lymphoid organs, whereas apoptotic cells mobilize passively through circulation, from where they are captured by marginal zone phagocytes [23].

In mice injected with cells alive (3 days before), proliferating 1H3.1 CD4 T cells in the spleen up-regulated CD69 and reduced CD62L expression (Fig. 3A), although to a lower extent than the positive controls of T cell activation. Interestingly, in B6 mice that received BALB/c apoptotic cells, proliferating 1H3.1 T cells did not increase CD69 nor down-regulate CD62L (Fig. 3A), and when re-stimulated with IEα_52–68_ ex vivo contained a significantly lower frequency of IFN-γ-secreting cells compared to animals treated with cells alive or with apoptotic cells plus CD40 mAb (Fig. 3B). We did not detect differences in the percentages of CD4 FoxP3^+^ 1H3.1 cells (≤2%, by FACS), or the number of IL-4-, IL-10-, IL-17- or TGF-β1-secreting cells in response to IEα_52–68_ (≤25 spots/ 10^6^ splenocytes, by ELISPOT, not shown), between splenocytes B6 mice injected 3 days before (or not, controls) with BALB/c splenocytes alive or apoptotic.

**Figure 4:**
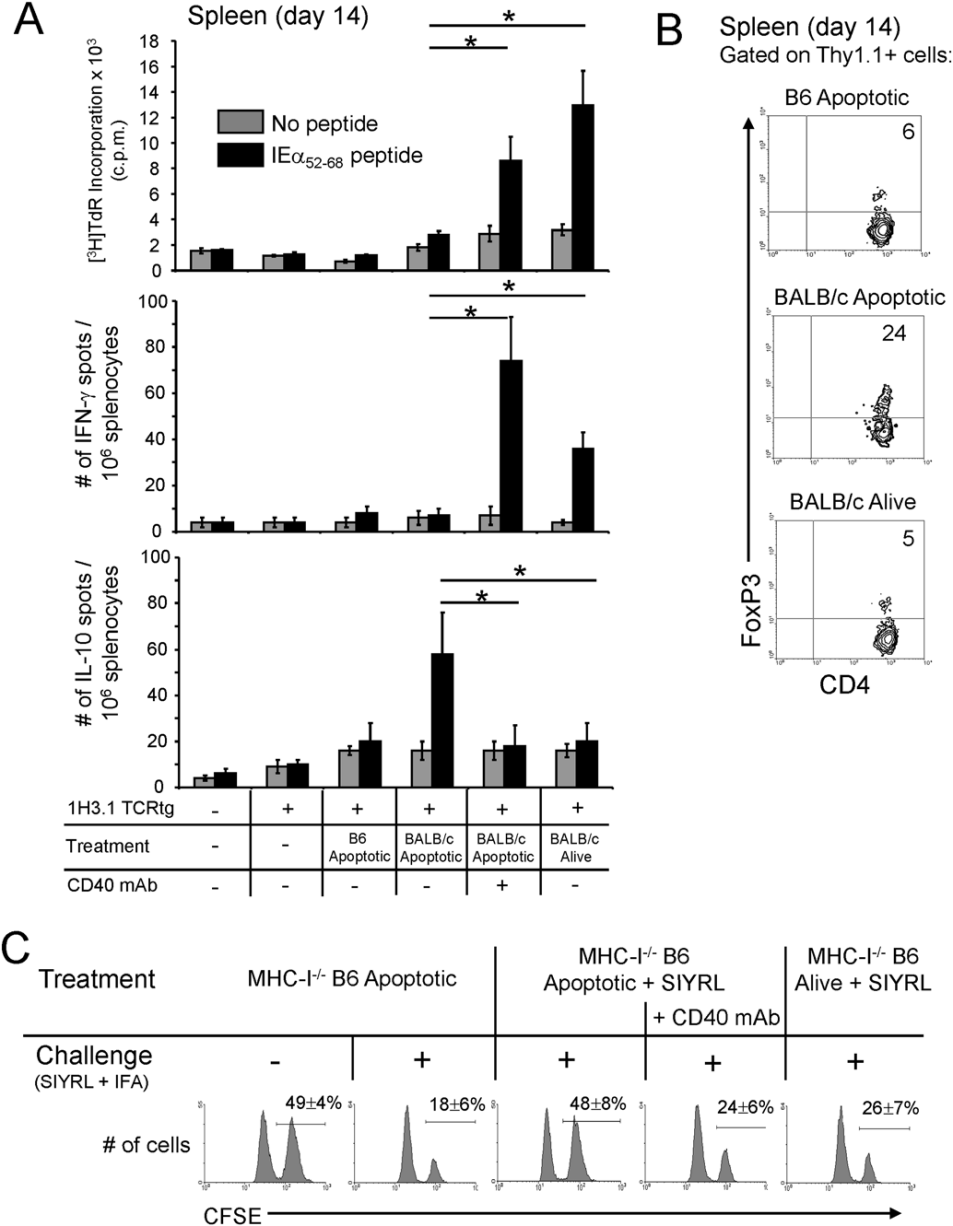
Administration of donor apoptotic cells regulates the indirect T cell responses. A) Allo-Ag presentation and ELISPOT assays of splenocytes from B6 mice reconstituted (or not, control) with 1H3.1 CD4 T cells and then injected i.v. with B6 (control) or BALB/c apoptotic splenocytes (alone or with agonistic CD40 mAb), or with BALB/c splenocytes alive. Fourteen days later, splenocytes from the host B6 mice were cultured alone or with the BALB/c peptide IEα_52–68_ during 72 h for assessment of T cell proliferation by [^3^H]TdR incorporation, or for 36 h in ELISPOT plates for quantification of IFN-γ and IL-10 secretion. Each group included 6 mice. B) Analysis by FACS of the percentage of CD4 1H3.1 T cells (Thy1.1^+^) expressing FoxP3 in the spleen of host B6 mice (Thy1.2^+^) 14 days after receiving i.v. B6 (control) or BALB/c apoptotic splenocytes, or BALB/c splenocytes alive. Numbers indicate percentages of cells. One representative of 6 individual experiments is shown. * p<0.01. C) Assessment by in vivo killing assays of the Ag-specific lytic activity of B6 mice reconstituted with 2C CD8 T cells and treated (day 1) with 10^7^ MHC-I^−/−^ B6 apoptotic or alive splenocytes loaded (or not) with the H2K^b^-retricted SYGL peptide. Mice were challenged (or not) on day 6 with SYGL plus IFA (i.p.). On day 9, mice were i.v. injected with a 1/1 mixture of CFSE^hi^ BALB/c×B6 (F1) (target) and CFSE^lo^ B6 (control) splenocytes. Five h later, the ratio of CFSE^hi^ vs. CFSE^lo^ cells in the spleen was determined by FACS. Numbers indicate percentages of cells. The specific lysis was calculated and is shown as the mean±SD of 3 mice per group.

The initial expansion in the percentage of splenic 1H3.1 T cells detected at day 3 in B6 mice treated with BALB/c cells alive or apoptotic, was followed at day 14 by a severe reduction in the number of 1H3.1 cells in mice treated with apoptotic cells (Fig. 3C). The finding that the percentage of 1H3.1 T cells in the lungs was lower (p<0.01) in mice treated with apoptotic cells compared to controls under CD40 stimulation or mice treated with cells alive, suggests that deletion instead of homing to periphery, was the cause of the reduction in the number of 1H3.1 T cells in mice that received apoptotic cells (Fig. 3C). At day 14, proliferation and the percentage of IFN-γ-secreting of 1H3.1 T cells in the spleen was lower (p<0.01) in mice injected with apoptotic splenocytes compared to animals treated with cells alive or to positive controls stimulated via CD40 (Fig. 4A). Interestingly, at day 14, B6 mice treated with BALB/c apoptotic cells exhibited an increase (p<0.01) in the percentage of splenic 1H3.1 T cell secreting IL-10 in response to IEα_52–68_ re-stimulation (Fig. 4A), and an augment in the percentage of FoxP3^+^ 1H3.1 T cells (Fig. 4B). In summary, systemic injection of donor apoptotic splenocytes, unlike donor cells alive, affects severely the development of type-1 indirect pathway CD4 T cells.

### Effect of apoptotic cell therapy on cross-priming of anti-donor CD8 T cells

Next, we tested if administration of apoptotic cells affects cross-priming of donor-reactive CD8 T cells in vivo. B6 mice, reconstituted with 2C TCRtg CD8 T cells (specific for SYGL-H2K^b^) [55], were treated the following day with apoptotic or alive β_2 m_^−/−^ B6 splenocytes loaded in vitro with SYGL peptide (10^7^ cells/ i.v.). Since these latter cells lack surface MHC class-I, the only mechanism by which the SYGL-H2K^b^ complex can be presented to 2C cells is through cross-priming by host's B6 (H2K^b^) APCs. After 6 days, B6 mice were challenged with SYGL peptide (plus IFA, i.p.), and three days later (day 9) the function of the transferred 2C cells was assessed by 5 h-in vivo killing assays. Given that our transplant model employs the BALB/c (donor)→B6 (recipient) combination, we selected B6×BALB/c (F1) splenocytes as targets, since (i) 2C cells also recognize H2L^d^ molecules (from BALB/c) (55), and (ii) the presence of “self-MHC molecules” (from B6) prevents killing of the target cells by host's NK cells. Administration of β_2 m_^−/−^ B6 apoptotic cells loaded with SYGL decreased the Ag-specific lytic activity of splenic 2C cells, when compared to controls that received apoptotic cells not exposed to the peptide (10±4 vs. 77±10% lysis, respectively, p<0.001) (Fig. 4C). This inhibitory effect was reversed by co-administration of agonistic CD40 mAb by the time of apoptotic cell infusion (67±8% lysis), and was not achieved by administration of the same number of β_2 m_^−/−^ B6 living splenocytes pulsed with SYGL (63±7% lysis) (Fig. 4C).

### Impact on development of CAV of in situ delivery of donor alloAg to recipient's DCs

**Figure 5:**
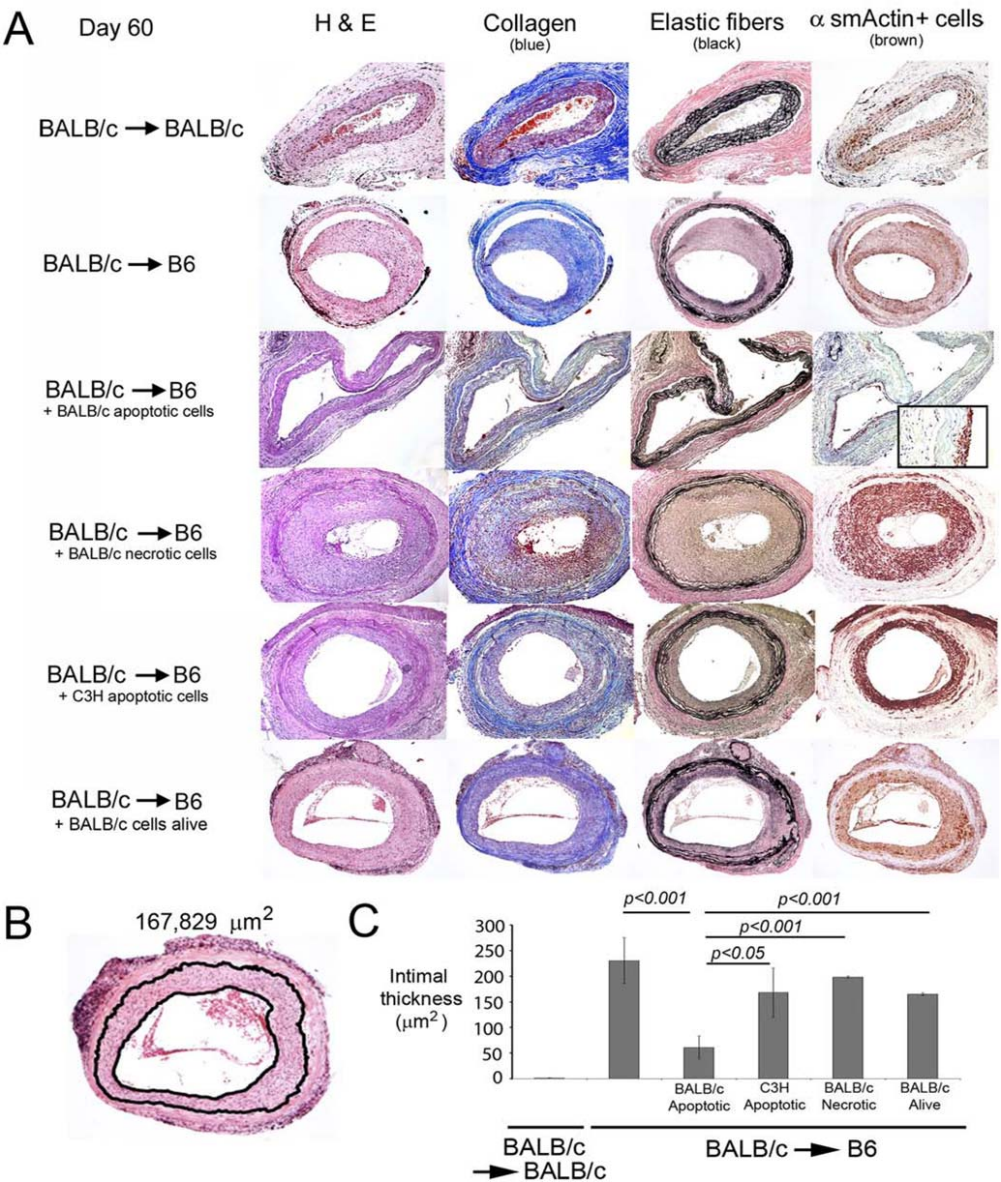
Effect of donor apoptotic splenocytes on CAV. A) Microscopic analysis of cross-sections of aortic BALB/c grafts procured 60 days after transplantation from syngeneic or allogeneic (B6) recipient mice left untreated, or injected (i.v., day -7) with BALB/c splenocytes apoptotic, necrotic or alive, or with C3H (third-party) apoptotic splenocytes. Sections were stained with H&E or to identify collagen deposition, elastic fibers, or α-sm actin^+^ cells. The sections shown are representative of 6 independent aortic transplants per group. B) Method employed to quantify morphometrically intimal thickening in aortic grafts by means of a microscope equipped with an imaging system and image analyzing software. C) Comparison of the intimal thickening of aortic BALB/c grafts, 60 days after transplantation in syngeneic or B6 recipient mice left untreated or injected with BALB/c splenocytes apoptotic, necrotic, or alive, or with C3H (third-party) apoptotic splenocytes. Results represented values pooled from 6 mice per group.

Next, we tested the effect of targeting in situ recipient's splenic DCs with donor apoptotic cells on development of CAV in a model of aortic allografts. B6 recipient mice were injected (i.v., d-7) with 10^7^ BALB/c apoptotic splenocytes, and then transplanted with BALB/c aortas. Controls included B6 mice untreated, or injected with BALB/c (donor) alive or necrotic, or C3H (third-party) apoptotic splenocytes (6 mice per group, Fig. 5A). The aortic allografts were examined 60 days after transplantation.

**Figure 6:**
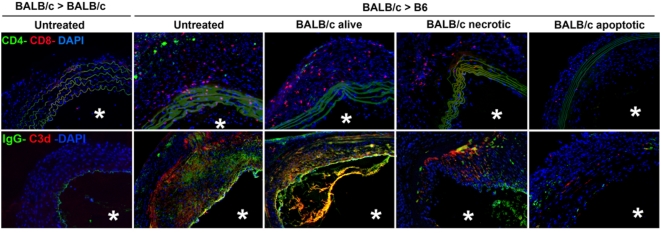
Impact of donor apoptotic splenocytes on lymphocyte infiltration and allo-Ab deposition in aortic grafts. Detection by fluorescence microscopy of infiltrating CD4 and CD8 T cells, and IgG and C3d deposited in aortic BALB/c grafts, 60 days after transplant in syngeneic (control) or B6 recipient mice, left untreated or injected with BALB/c splenocytes, apoptotic, necrotic, or alive. Cell nuclei were stained blue with DAPI. Asterisks indicate the vessel lumen. ×200.

No histopathological changes were detected in control BALB/c aortas transplanted in syngeneic recipients and were indistinguishable from native BALB/c aortas (Fig. 5A). BALB/c aortic grafts transplanted in untreated B6 mice showed severe signs of CAV, including: (i) diffuse intimal thickening comprising the entire circumference of the artery and caused mainly by infiltration of α-sm actin^+^ cells; (ii) partial fragmentation and thickening of the internal elastic membrane; (iii) deposition of collagen; and (iv) infiltration by CD4 and CD8 T cells (Fig. 5A–C and Fig. 6). Compared to allografts from untreated mice, BALB/c aortas from B6 recipients treated with donor apoptotic cells exhibited the following features: (i) significant reduction of intimal thickening (p<0.001); (ii) minimum infiltration of α-sm actin^+^ cells; (iii) no damage of the elastic fibers; (iv) substantial reduction in the amount of collagen deposition; and (v) decreased number of infiltrating CD8 and CD4 T cells (Fig. 5A–C and Fig. 6). We did not detect CD4 Foxp3^+^ T cells within the allografts.

The beneficial effect of donor apoptotic cells on CAV was dependent on the intrinsic properties of the apoptotic cells, since the histopathology of aortic allografts from controls treated with donor splenocytes alive or necrotic was similar to that of untreated mice (Fig. 5A). The effect of apoptotic cell therapy on CAV was donor-specific, since injection of third-party (C3H) splenocytes in early apoptosis did not have a significant effect on the parameters of CAV analyzed (Fig. 5A).

### The beneficial effects of donor apoptotic cells on CAV are associated to down-regulation of the anti-donor response

**Figure 7:**
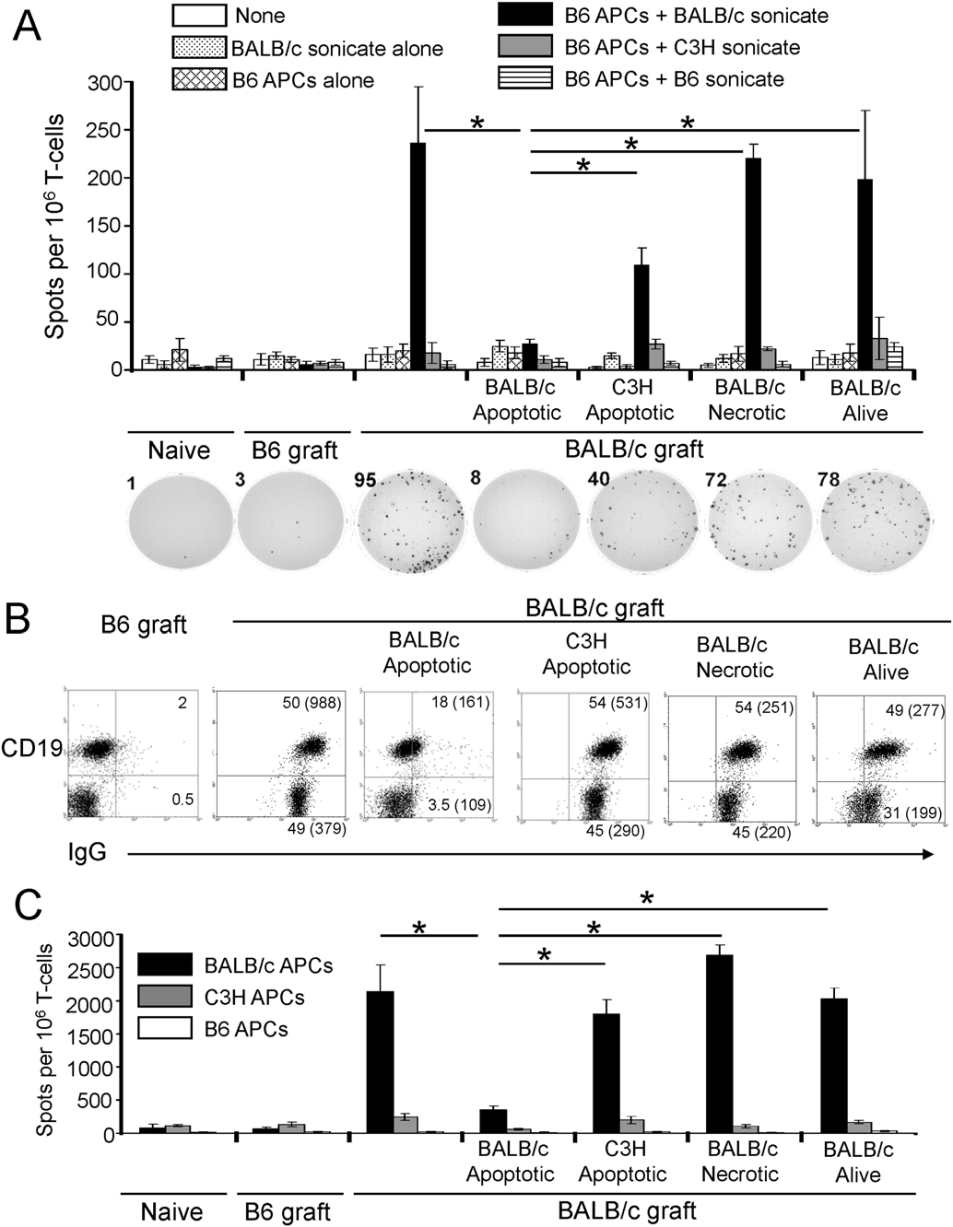
Regulation of the systemic anti-donor response by donor apoptotic splenocytes. A) Assessment by IFN-γ ELISPOT assay of the indirect T cell response elicited in the spleens of B6 mice, naïve (control), or transplanted 60 days before with syngeneic (control) or BALB/c aortic grafts. Numbers indicate the number of spots. Each group included 4 mice. B) Detection by FACS of circulating alloAb in B6 recipients of syngeneic or BALB/c aortic grafts under different conditions and analyzed 60 days after surgery. Results are representative of 6 mice per group. Numbers indicate percentages of cells. Numbers in parenthesis represent mean fluorescence intensity. C) Evaluation of the direct T cell response by IFN-γ ELISPOT assay in the spleen of B6 mice, naïve (control) or 60 days after being transplanted with syngeneic (control) or BALB/c aortas, under different conditions. Each group included 4 mice per group. * p<0.001.

We hypothesized that presentation of donor alloAgs by recipient's quiescent DCs controls the indirect anti-donor response, key in the pathogenesis of CAV [10]–[12]. Therefore, we assessed the effect of donor apoptotic cell therapy on the indirect T cell response, 60 days after transplantation of BALB/c aortas in B6 recipients. The frequency of responder splenic T cells (purity ≥96%) secreting IFN-γ following stimulation with splenic B6 APCs plus sonicates from BALB/c (donor), C3H (third-party) or B6 (syngeneic) splenocytes was evaluated by ELISPOT. The frequency of spots for IFN-γ was significantly reduced (p<0.001) in recipients that received donor apoptotic cells, when compared to mice left untreated or injected with BALB/c splenocytes alive or necrotic, or with C3H apoptotic cells (Fig. 7A). Responder T cells did not release IFN-γ when incubated with BALB/c sonicates alone, indicating that the sonicates were sufficiently disrupted to prevent any direct allorecognition, and that there were not residual recipient's APCs in the purified responder T cells.

Since delivery of donor alloAgs to recipient's DCs in vivo with donor apoptotic cells down-regulated the indirect T cell response, we next tested if this mechanism also affected the downstream cognate interaction between indirect Th cells and donor-reactive B cells, and the subsequent production of allo-Abs. Sixty days after aortic transplantation, recipients treated with donor apoptotic splenocytes decreased drastically the levels of circulating total IgG (Fig. 7B), IgG1 and IgG2a (not shown) allo-Abs. The reduction in the levels of allo-Abs in sera of recipients treated with donor apoptotic cells was not caused by deposition of the allo-Abs in the grafts. Microscopic analysis demonstrated that aortic allografts from recipients treated with donor apoptotic cells exhibited considerably lower amounts of locally deposited IgG (end-titer 1∶25) and C3d compared to grafts from recipients untreated (IgG end-titer 1∶400), or injected with BALB/c splenocytes alive (IgG end-titer 1∶400) or necrotic (IgG end-titer 1∶200).

Next, we analyzed if therapy with donor apoptotic cells affected the direct T cell response. Sixty days after transplantation of BALB/c aortas, the frequency of (B6) splenic T cells secreting IFN-γ in response to splenic BALB/c, B6 or C3H APCs was assessed by ELISPOT. Administration of donor apoptotic cells decreased significantly (p<0.001) the type-1 direct T cell response compared to controls, in a donor-specific fashion, as demonstrated by the direct response against third-party (C3H) APCs (Fig. 7C).

## Discussion

In the present study, we demonstrated that delivery of donor allo-Ags to recipient's DCs in situ is an efficient approach to control the indirect anti-donor response that contributes to CAV, a key component of CR. Previous studies have shown the possibility of down-regulating the T cell response by delivering model Ags to quiescent DCs resident in secondary lymphoid organs [56]–[59]. In mice, this have been accomplished by administration of antigenic peptides linked to CD205-mAb, which recognizes the lectin DEC205 expressed by DCs of T cell areas in secondary lymphoid organs [56]–[59]. For transplantation, this elaborated technique may result impractical for in situ-delivery of the entire repertoire (or only the immuno-dominant) allo-peptides to recipient's DCs, due to haplotype variability in each donor-recipient combination. In recent years, delivery of donor allo-Ags to recipient DCs is situ has been achieved by administration of apoptotic cells [23], [37], [38] or DC-derived exosomes [60].

A complication to investigating the impact of delivery of foreign Ag to DCs in vivo and the resulting outcome on the T cell response is the existence of different DC subsets whose roles in peripheral tolerance are still unclear. pDCs could potentially mediate the regulatory effect of donor apoptotic cells on anti-donor T cells, since they facilitate transplantation tolerance in mice [45]–[47]. However, previous studies have not addressed the role of pDCs in the regulation of the T cell response that follows apoptotic cell therapy, and the capability of pDCs to phagocytose apoptotic cells is still controversial. Dalgaard et al. [48] have demonstrated that pDCs are unable to phagocytose apoptotic cells, whereas others have shown that pDCs internalize fluorochromes, latex beads, immuno-complexes and virus-infected apoptotic cells [49]–[51]. Our findings indicate that splenic CD11^hi^ DCs (CD8α^+^ and CD8^−^), but not pDCs, are targeted by i.v. injection of apoptotic cells, which agrees with the previous findings that splenic CD8α^+^ DCs are responsible for at least some of the beneficial effects of apoptotic cell therapy [37], [61].

Internalization of apoptotic cells down-regulates the T cell stimulatory function of DCs by reducing their expression of MHC and costimulatory molecules and secretion of pro-inflammatory cytokines, while augmenting release of anti-inflammatory IL-10 and TGF-β1 [23], [26]–[31]. This is due to the presence on the apoptotic cell surface of Apoptotic Cell Associated Molecular Patterns (ACAMPs), which are recognized as “eat me” signals by pattern recognition receptors on the surface of phagocytes during the assembly of the phagocytic synapse [62]. The ACAMPs dock the apoptotic cell to the phagocyte and deliver inhibitory signals to the APCs, that are critical to prevent autoimmunity following apoptotic cell clearance of self-tissues in physiological conditions. Therefore, apoptotic cells constitute an ideal vehicle to deliver in situ simultaneously donor allo-Ags and inhibitory signals to DCs of graft recipients, and thus down-regulate indirect allorecognition. Our findings demonstrate that DCs that have endocytosed donor apoptotic cells in vivo not only retain a low ratio of co-stimulatory: inhibitory signals but also resist ex vivo-maturation in response to DC-activating signals. Our results also show that, unlike macrophages [63], those DCs affected by the inhibitory effects of apoptotic cells do not exert bystander effect on DCs that did not take up apoptotic cells.

Our study has addressed an important question for future development of efficient apoptotic cell-based therapies for promotion of transplantation tolerance: *for how long recipient DCs are able to present apoptotic cell-derived allopeptides to donor-reactive T cells following systemic administration of donor apoptotic cells?* Although splenic DCs live between 1.5–2.9 days [64], indirect evidence indicates they may present Ag for as long as 14 days [65]. This discrepancy has been explained partly by the observation that proliferating DCs prolong Ag presentation through passive transfer of long-lived peptide-MHC complexes to daughter DCs [65], [66]. However, our finding that presentation of donor apoptotic cell-derived allopeptides declines 3 days after apoptotic cell infusion indicates that long-term presentation of donor allo-Ag for induction/maintenance of transplant tolerance may require repetitive administration of apoptotic cells.

We hypothesize that following in situ targeting with donor apoptotic cells, recipient's quiescent DCs of the spleen will present the donor peptides in a pro-tolerogenic fashion due to the inhibitory signals delivered by the apoptotic cells. However, in theory, administration of donor splenocytes alive should exert a similar effect, since the allogeneic cells will eventually die or become targets of recipient's NK cells. Surprisingly, numerous reports on the immunosuppressive effects of apoptotic cells did not include living cells as controls [21], [39]–[41], and studies on the beneficial effects of systemically administered donor splenocytes alive (donor specific transfusion, DST) in transplantation did not compare their results with controls injected with donor apoptotic splenocytes [67], [68]. In our model, although administration of donor splenocytes alive or apoptotic triggered initial expansion of indirect CD4 T cells, only apoptotic cell infusion was associated with deficient activation and deletion of indirect T cells. Interestingly, those T cells that escaped deletion after apoptotic cell infusion exhibited reduced percentages of IFN-γ-secreting cells and increased numbers of IL-10-releasing cells and FoxP3^+^, compared with controls treated with donor splenocytes alive. These results agree with previous findings by us [37] and others [69] that systemic administration of apoptotic cells bearing allo-Ags or haptens is associated with generation/expansion of donor- or hapten-specific Treg in mice. The regulatory effects of donor apoptotic cells on indirect CD4 T cells explains their therapeutic potential on CAV, since in murine and pig models, CAV is initiated by CD4 T cells recognizing allo-Ag via the indirect pathway [10]–[12], [70].

Previous studies have shown that DST, particularly when combined with CD40-CD40L (CD154) blockade, promotes tolerance in cardiac allografts in mice [67], [68]. However, in our model and unlike donor apoptotic cell therapy, DST alone did not prevent development of CAV. It has been proposed that a generalized immune-suppressive effect results from the transfusion to the recipient of a large number of phosphatidylserine bearing cells, which through interaction with phosphatidylserine receptors expressed by phagocytes results in down-regulation of the stimulatory function of APCs [71]. Likely, in our system, the fact that apoptotic cell death was synchronized by UVB-irradiation and the apoptotic cells were administered as an i.v. bolus could explain their potent immune-regulatory effect s in vivo. A similar phenomenon could be the reason of the beneficial effects on allograft rejection [72], [73] and GVHD [74] of extracorporeal photopheresis, a method that re-infuses elevated numbers of UV-induced apoptotic PBMCs. Interestingly, tolerance associated to therapy with depleting CD3 mAb is due to the regulatory effects on APCs caused by a high number of apoptotic T cells generated synchronically in vivo [75]. Unlike donor apoptotic cell therapy, following DST: (i) generation of apoptotic cells in vivo is asynchronic with slower kinetics and probably less inhibitory potential on recipient APCs; and ii) recipient APCs may receive CD40-mediated stimulation through CD154 expressed by transfused allo-reactive CD4 T cells. The synergistic effect of CD40-CD154 blockade on DST is likely due to (i) its pro-apoptotic effects on adoptively transferred donor cells, since CD40-signalling promotes survival of B lymphocytes and DCs; and (ii) blockade of CD40-mediated activation of recipient DCs presenting donor Ag acquired from the DST. Interestingly, both effects can be achieved by simple administration of donor cells in early apoptosis.

Our results indicate that down-regulation of the indirect alloresponse by donor apoptotic cell therapy prevents/ameliorates CAV in aortic allografts, an established model of chronic rejection elicited through the indirect pathway. Thus, development of CAV in aortic grafts constituted an elegant model to investigate the effect of donor apoptotic cell therapy on the indirect alloresponse. Compared to alternative models of CAV in coronary arteries, aortic grafts (i) facilitate the morphological analysis of the anti-donor response within the vessels due to their bigger size, and (ii) are not affected by the inflammation in the surrounding tissue, as occurs in coronary arteries during cardiac allograft rejection. Histologically, the artery allografts of animals treated with donor apoptotic cells, did not have luminal occlusion and exhibited slight intimal thickening with few α sm actin^+^ cells, no damage of elastic fibers, substantially less collagen accumulation, and reduced T cell infiltration. These beneficial effects on CAV were allo-specific and dependent on the physical properties of the apoptotic bodies, since administration of donor leukocytes alive or necrotic did not affect the histological signs of CAV. Importantly, analysis of the indirect allo-response 60 days post-transplant, confirmed that only those animals treated with donor apoptotic cells exhibited reduced type-1 T cell anti-donor responses and allo-Ab secretion.

Interestingly, our separate analysis of the direct and indirect anti-donor responses in recipients treated with donor apoptotic cells has revealed that the proposed model of the mechanism of action of apoptotic cell therapy in transplantation hides an important paradox: *if donor apoptotic cells are required to be re-processed by recipient APCs to down-regulate indirect pathway T cells, why donor apoptotic cell therapy also reduces the anti-donor T cell response elicited through the direct pathway?* This phenomenon explains why apoptotic cell therapy promotes heart allograft survival in murine models where the direct pathway plays a dominant role in acute rejection [37], [38]. This paradoxical effect could be caused by: (i) interaction of the donor apoptotic cells with direct pathway T cells or, more likely, (ii) down-regulation of the indirect CD4 T cell help (through T cell deletion or generation of Treg) required for maturation of direct T cells into effector cells [76], [77].

Development of novel methodologies to control the anti-donor T cell response elicited through the indirect pathway represent an effective strategy to ameliorate development of CAV and CR, the major obstacles to the long-term success of solid organ transplantation that the currently employed immunosuppressive drugs fail to prevent. In situ-delivery of the entire repertoire of donor alloAg along with a potent inhibitory signal to recipient's DCs in secondary lymphoid organs through donor-derived apoptotic bodies represents a novel, simple and relatively safe approach to down-regulate the indirect alloresponse to achieve that goal.
